# Association between experiences of intimate partner sexual violence and cigarette smoking among women in union in Papua New Guinea: evidence from a nationally representative survey

**DOI:** 10.1186/s12889-022-13003-4

**Published:** 2022-03-29

**Authors:** Bernard Yeboah-Asiamah Asare, Williams Agyemang-Duah, Emmanuel Brenyah Adomako, Parul Puri, Deborah Odunayo Ogundare, Deepanjali Vishwakarma, Prince Peprah

**Affiliations:** 1 Curtin School of Population Health, Curtin University, Kent Street, 6102 Perth, Australia; 2 Health Psychology, Institute of Applied Health Sciences, University of Aberdeen, AB25 2ZD Aberdeen, UK; 3 Department of Geography and Planning, Queen’s University, Kingston, ON K7L 3N6 Canada; 4 Social Work Department, School of Health and Society, University of Wollongong, Wollongong, Australia; 5 Department of Survey Research and Data Analytics, International Institute for Population Sciences, Mumbai, 400088 Maharashtra India; 6 Nigeria Centre for Disease Control, Abuja, Nigeria; 7 Centre for Primary Health Care and Equity/ Social Policy Research Centre, University of New South Wales, Sydney, Australia

**Keywords:** Demographic and health survey, Intimate partner sexual violence, Cigarette smoking, Papua New Guinea

## Abstract

**Background:**

Intimate partner sexual violence (IPSV) is a prevalent public health problem affecting millions of people each year globally, particularly in developing countries like Papua New Guinea (PNG). Although over two-thirds of women in PNG are estimated to experience some form of sexual violence in their lifetime, empirical evidence is limited on the association between IPSV and cigarette smoking. Thus, the present study aims to examine the prevalence of IPSV and its association with cigarette smoking among women in union  in PNG.

**Methods:**

This cross-sectional study used data from the first demographic and health survey of PNG conducted between 2016 and 2018. A total of 9,943 women aged 15–49 years in intimate unions were included in this study. We estimated the relative risk of smoking cigarette using modified Poisson regression models with a robust variance and 95% confidence intervals.

**Results:**

The rates of IPSV and current cigarette smoking were 25.9% and 26.8%, respectively. The modified Poisson regression results showed that IPSV was significantly associated with an elevated risk for cigarette smoking. Women with IPSV history were more likely to smoke cigarette relative to their counterparts with no IPSV history (RR: 1.33, 95% CI: 1.18–1.50) in the absence of covariates. After controlling for demographic, social and economic factors, the association between IPSV and cigarette smoking remained statistically significant (RR: 1.24, 95% CI: 1.08–1.42).

**Conclusions:**

The rates of IPSV and cigarette smoking among women in union in PNG in the current study were relatively high. Irrespective of diverse demographic, social and economic factors, IPSV was still significantly associated with cigarette smoking among women in union in PNG. The findings presented call the attention of policy-makers and relevant authorities in PNG to an important association that needs to be addressed. Counseling, awareness creation, service provision and program design on IPSV are urgently required to minimize cigarette smoking and IPSV among women in union in PNG.

**Supplementary Information:**

The online version contains supplementary material available at 10.1186/s12889-022-13003-4.

## Background

Globally, intimate partner sexual violence (IPSV), a form of intimate partner violence (IPV) has been declared as a public health issue considering its long-term physical, biological, psychological and neurological consequences on victims in society [1–3]. IPSV is defined as sexual acts committed or attempted by an intimate partner without the consent of the victim or against someone unable to give consent [4]. It involves rape, unwanted pressured penetration, intentional sexual touching, and non-contact acts of sexual nature [4]. IPSV is common among women globally although, some men could also be victims; an estimated 1 out of 10 men experience IPSV while 1 out of 4 women experience IPSV in their lifetime [4]. Also, a recent literature review reported that the prevalence of IPSV among women range from 9.4% -15.2% in several countries in the Americas [5].

Several factors have been indicated to contribute to high levels of IPSV among women. For instance, it is established that cohabiting partners report higher rate of sexual violence compared with married women [5]. Furthermore, socio-cultural beliefs of women being recognized as the property of men, and other socio-demographic characteristics such as poverty, financial insecurity, lower level of education, and smoking among others have also been documented to be associated with IPSV [1, 2, 4–6].

 Cigarette smoking is indicated to be prevalent among women who are victims of IPSV [4, 7]. Several studies have established sexual violence to be one of the significant predictors of cigarette smoking among women [2, 7, 8]. Smoking is assumed to ease the stress women undergo in abusive relationships [1, 2, 4]. A longitudinal study has found that women who smoke are likely to report sexual violence compared to women who do not smoke [4].

Papua New Guinea (PNG) is known to have high prevalence of sexual violence against women [9, 10]. About 41% of men admitted raping a woman while one third of women have suffered sexual violence [10]. Population-based studies in PNG have demonstrated high prevalence of IPSV and IPV in general [9, 10], however they have scarcely considered the influence of IPSV on cigarette smoking among women in union. Thus, irrespective of the high prevalence of intimate partner violence in PNG studies linking IPSV and cigarette smoking among women in union are limited. This study extends the present literature by examining the association between IPSV and current cigarette smoking among women in union in PNG. This is an important public health issue for many reasons. While IPSV has been associated with an elevated risk for physical and mental health problems, cigarette smoking increases the risk of adverse health outcomes. The aim of the present study is to examine the prevalence of IPSV and its association with cigarette smoking among women in union in PNG by controlling for demographic, social and economic factors. Thus, the study seeks to test whether IPSV is significantly associated with cigarette smoking and the role of socio-demographic and economic factors in the association. This study focused on sexual violence because it is one of the most prevalent forms of gender-based violence in PNG [10]. Furthermore, some studies have suggested that while sexual violence may include physical violence, factors associated with each violence form could differ and as a result it is imperative to separately focus on each form of IPV [1, 11, 12].

## Methods

### Data source, sampling technique and sample size

This cross-sectional study used data from the Papua New Guinea Demography and Health Survey (PNGDHS) conducted from October 2016 to December 2018. This is the first demographic and survey conducted in PNG. The PNGDHS aimed to generate comprehensive data on demographic, maternal and reproductive issues such as fertility, family planning awareness and practices, breastfeeding practices, health behaviors, immunizations, domestic and intimate partner violence, among others. Through the Demographic and Health Survey (DHS) programme, technical support for the execution of the survey was provided by Inner City Fund (ICF), with the financial support of Papua New Guinea Government, Australian Government Department of Foreign Affairs and Trade, the United Nations Population Fund (UNFPA) and UNICEF [13]. The sample for the 2016–18 PNGDHS was nationally representative and covered the entire population that lived in private dwelling units in the country. The survey used the list of census units (CUs) from the 2011 Papua New Guinea National Population and Housing Census as the sampling frame and adopted a probability-based sampling approach. Specifically, a two-stage stratified cluster sampling procedure was followed. The methodology and selection procedure details have been reported in the PNGDHS final report.

In summary, each province in the country was stratified into urban and rural areas, yielding 43 sampling strata, except the National Capital District, which has no rural areas. The division paid particular attention to urban–rural variations. Samples of census units were selected independently in each stratum in two stages. In the first stage, sorting the sampling frame within each sampling stratum to achieve implicit stratification and proportional allocation using a probability proportional-to-size selection was done. In the second stage of sampling, a fixed number of 24 households per cluster were selected with an equal probability systematic selection from the newly created household listing, resulting in a total sample size of approximately 19,200 households. To prevent bias, no replacements and no changes of the pre-selected households were allowed in the implementing stages. In cases where a census unit had fewer than 24 households, all households were included in the sample. A total of 17,505 households were selected for the sample, of which 16,754 were occupied. Of the occupied households, 16,021 were successfully interviewed, yielding a response rate of 96%. In the interviewed households, 18,175 women age 15–49 were identified for individual interviews; interviews were completed with 15,198 women, yielding a response rate of 84%. In this present study, the sample comprised 9,943 women who were in union (either married or cohabiting) during the survey. Thus, our analysis used data only on women who were in union during the survey.

### Data availability and ethical consideration

The data have been archived in the public repository of DHS. The access to the data requires registration which is granted specifically for legitimate research purposes. Consent forms were administered at household and individual levels, in accordance with the Human Subject Protection. The dataset can be accessed at https://dhsprogram.com/data/dataset/Papua-New-Guinea_Standard-DHS_2017.cfm?flag = 0.

### Main outcome and predictor variables

Current cigarette smoking was the outcome variable in this study. This was measured as having smoked cigarette in the last 24 h before the survey. Women in union were asked the question: Smoked cigarette in the last 24 h? Women in union current smoking status were classified as “No” (0): no current smoking in the last 24 h or “Yes” (1): smoking in the last 24 h. The key explanatory variable in this study was IPSV. This variable was derived from the optional domestic violence module, where questions are based on a modified version of the conflict tactics scale [14, 15]. Questions asked are in relation to physical, sexual or emotional violence experiences. In this study, the focus was on the experience of sexual violence. Three standard items including whether the partner ever physically forced the respondent into unwanted sex; whether the partner ever forced respondent into other unwanted sexual acts and; whether the respondent has been physically forced to perform sexual acts she did not want to were used to generate the experience of intimate partner sexual violence. For each of these items, the responses were ‘never’ ‘often’ ‘sometimes’ and ‘yes, but not in the last 12 months. However, for our analysis purpose, we created a dichotomous variable to represent whether a respondent had experienced sexual violence in the past 12 months. This was done by recoding the following responses: ‘never’ and ‘yes, but not in the last 12 months’ as “No” (0) and ‘yes’, ‘often’ and ‘sometimes’ as “Yes” (1).

### Confounding variables

Theoretically and empirically relevant demographic and socioeconomic variables were included as confounders. In all, we included twenty socioeconomic and demographic variables to adjust for in the modelling. These variables included age, region, religion, place of residence, highest educational level, literacy, marital status, residing with a partner, number of partner’s wives, partner’s age, partner’s education, health insurance cover, internet access, mobile phone ownership, watch television, listen to radio, read newspaper/magazine, occupation and wealth index. The selection of these variables was informed by their statistically significant associations with sexual violence and cigarette smoking in previous studies [1, 2, 4, 16, 17]. (See Table 1 for the details on the coding of the covariates).

### Statistical analysis

Before the analysis, all missing data were removed. Both descriptive (frequencies, percentages, mean and standard deviation) and inferential (chi-square and modified Poisson regression) analytical frameworks embedded in STATA software version 13.0 (StataCorp LP, College Station, Texas, USA) were used. The statistical analysis followed some essential steps. We performed descriptive statistics such as frequencies and percentages to describe the sample. The Pearson’s Chi-square test was done to examine the differences in smoking cigarette by socio-demographic characteristics and IPSV. A modified Poisson regression, adjusting for demographic, social and economic variables, was also performed to model the association between IPSV and cigarette smoking, to estimate the relative risk (RR) of cigarette smoking [18, 19].

The study used the modified Poisson regression that incorporates the robust error variance procedure to optimize the accuracy of the estimates [18], as direct estimates of relative risk produce from modified Poisson regression modelling may be a preferred method for estimating population-level risk [19]. We fitted four regression models. Model 1 included dependent and independent variables only; thus, was the base model. While adjusting for the theoretically relevant confounding variables, Models 2, 3 and 4, respectively introduced demographic and socioeconomic factors to investigate whether these variables play any role and might tamper the effects of IPSV on cigarette smoking. Before the regression analysis, diagnostics checks for multicollinearity were conducted using the variance inflation factor (VIF). In this analysis, none of the VIF scores exceeded the value of 2.38, suggesting no multicollinearity. The results of the regression analyses were presented as crude relative risk (CRR) and adjusted relative risk (ARR) at 95% confidence intervals (CIs). All the estimates provided in this study are derived by applying appropriate sampling weights supplied by PNGDHS, 2016–18. A statistical significance threshold of *p* ≤ 0.05 was selected.

## Results

### Background characteristics of the participants

The mean age of the participants was 32.68 ± 0.08 years. About 20% of the participants were aged between 25–29 years, 28.6% were from the Highlands Region, 75.5% resided in the rural area, 49.7% had primary level of education, and 99% were Christians. Again, 83% of the participants were married, 86.6% were currently living together with their partner, and 33.8% had between 1–2 kids and 62.1% were not working (see Table 1).

**Table 1:** Background characteristics of respondents

Characteristics	Total n(%)	Cigarette smoking		Pearson chi-square
		**Yes n (%)**	**No, n (%)**	***P*-value**
**Age groups (yrs)**				< 0.001
15–19	358(3.6)	71(19.8)	287(80.2)	
20–24	1493(15.1)	357(23.9)	1136(76.1)	
25–29	2008(20.4)	522(26.0)	1486(74.0)	
30–34	1900(19.3)	457(24.0)	1443(76.0)	
35–39	1755(17.8)	353(20.1)	1402(79.9)	
40–44	1324(13.4)	271(20.5)	1053(79.5)	
45–49	1030(10.4)	117(17.2)	853(82.8)	
**Region**				< 0.001
Southern	2810(28.5)	509(18.1)	2301(81.9)	
Highlands	2817(28.6)	647(23.0)	2170(77.0)	
Momase	2014(20.4)	579(28.8)	1435(71.3)	
Islands	2227(22.6)	473(21.2)	1754(78.8)	
**Place of residence**				< 0.001
Rural	7453(75.5)	1485(19.9)	5968(80.1)	
Urban	2415(24.5)	723(29.9)	1692(70.1)	
**Highest education level**				0.009
No education	2266(23.0)	530(23.4)	1736(76.6)	
Primary	4899(49.7)	1028(21.0)	3871(79.0)	
Secondary	2303(23.3)	559(24.3)	1744(75.7)	
Higher	400(4.0)	91(22.8)	309(77.2)	
**Religion**				0.003
Christian	9756(99.0)	2170(22.2)	7586(77.8)	
Non-Christian	53(0.6)	17(32.1)	36(67.9)	
No religion	47(0.4)	19(40.4)	28(59.6)	
**Wealth index**				< 0.001
Poorest	1485(15.0)	340(22.9)	1145(77.1)	
Poorer	1576(16.0)	340(21.6)	1236(78.4)	
Middle	1835(18.6)	334(18.2)	1501(81.8)	
Richer	2393(24.3)	518(21.7)	1875(78.4)	
Richest	2579(26.1)	676(26.2)	1903(73.8)	
**Marital status**				0.080
Married	8193(83.0)	1806(22.0)	6387(78.0)	
Co-habitation	1675(17.0)	402(24.0)	1273(76.0)	
**Currently residing with partner**				0.031
Living together	8503(86.6)	1867(22.0)	6636(78.0)	
Staying elsewhere	1316(13.4)	324(24.6)	992(75.4)	
**Number of kids**				< 0.001
None	945(9.6)	272(28.8)	673(71.2)	
1–2	3335(33.8)	792(23.8)	2543(76.2)	
3–4	3140(31.8)	688(21.9)	2452(78.1)	
5–6	1734(17.6)	315(18.2)	1419(81.8)	
7 and more	714(7.2)	141(19.8)	573(80.2)	
**Occupational status**				< 0.001
Not working	6044(62.1)	1348(22.3)	4696(77.7)	
Professional/technical/managerial	570(5.9)	112(19.7)	458(80.3)	
Clerical	210(2.2)	55(26.2)	155(73.8)	
Sales	486(5.0)	123(25.3)	363(74.7)	
Agricultural	1508(15.5)	261(17.3)	1247(82.7)	
Services	841(8.6)	254(30.2)	587(69.8)	
Manual job	77(0.8)	23(29.9)	54(70.1)	
**Sexual violence**				< 0.001
No	2688(74.1)	551(20.5)	2137(79.5)	
Yes	938(25.9)	251(26.8)	687(73.2)	

#### Distribution of cigarette smoking across exposure to IPSV

Table 1 shows the distribution of cigarette smoking across IPSV. The results showed significant disparities in cigarette smoking and IPSV at *p* < 0.001. Specifically, 25.9% of women were exposed to IPSV while 26.8% of women who were exposed to IPSV smoked cigarette.

#### Association between exposure to IPSV and current cigarette smoking among women in union in PNG

Table 2 shows the results of the association between IPSV and current cigarette smoking among women in union in PNG. We found in all the four models that women who were exposed to IPSV were more likely to smoke cigarette. In Model 1, the study revealed that those who had experienced IPSV have a higher risk of smoking cigarette compared to those who had not experienced IPSV. In Model II, demographic variables were added to the variable in Model I, the study revealed that those who had experienced IPSV, those from the Highlands region, those residing in urban areas, those with no religion and those whose partners have 3 or more wives have a higher risk of smoking cigarette compared with their counterparts. Also, participants aged 45–49 years and those who are able to read a whole sentence have a lower risk of smoking cigarette compared to their counterparts. In Model III, when other social variables were added to all variable in Model II, the study revealed that those who had experienced IPSV, those residing in the Momase region, those living in urban areas, those with no religion and those whose partners have 3 or more wives have a higher risk of smoking cigarette compared with their counterparts. Again, participants aged 45–49 years and those who are able to read a whole sentence have a lower risk of smoking cigarette compared with their counterparts. In the final Model (Model IV), when economic variables were added to all variables in Model III, the study revealed that those who had experienced IPSV, those from the Momase region, those with no religion, those residing in urban area and those whose partners have 3 or more wives have a higher risk of smoking cigarette compared with their counterparts. Further, participants aged 45–49 years, those who rated their wealth index as ‘Middle’, those who were Clerical officers and those who are able to read a whole sentence have a lower risk of smoking cigarette (see Table 2). The key take home message from the result is that, after adjusting for diverse demographic, social and economic characteristics at different levels of the Model(s), IPSV remained significantly associated with cigarette smoking among the participants as the direction of the association remained.

**Table 2:** A modified Poisson regression of the relationship between IPSV and current smoking status

**Predictors**	Model ICrude RR (95%CI)	Model II Adjusted RR (95%CI)	Model III Adjusted RR (95%CI)	Model IV Adjusted RR (95%CI)
**Experience sexual violence**				
No	1.00	1.00	1.00	1.00
Yes	1.33(1.18–1.50)***	1.24(1.09–1.43)**	1.24(1.08–1.42)**	1.24(1.08–1.42)**
**Age groups (yrs)**				
15–19		1.00	1.00	1.00
20–24		0.91(0.62–1.34)	0.90(0.61–1.34)	0.92(0.62–1.36)
25–29		0.96(0.64–1.44)	0.96(0.64–1.46)	0.97(0.64–1.47)
30–34		0.89(0.59–1.35)	0.89(0.58–1.36)	0.91(0.59–1.38)
35–39		0.76(0.49–1.17)	0.76(0.49–1.18)	0.76(0.49–1.18)
40–44		0.64(0.40–1.01)	0.63(0.39–1.00)	0.64(0.40–1.02)
45–49		0.50(0.30–0.84)**	0.52(0.31–0.88)*	0.53(0.32–0.90)*
**Region**				
Southlands		1.00	1.00	1.00
Highlands		1.47(1.21–1.79)***	1.48(1.23–1.81)***	1.47(1.20–1.80)***
Momase		1.81(1.50–2.19)***	1.82(1.50–2.21)***	1.79(1.48–2.18)***
Islands		1.40(1.14–1.72)**	1.40(1.14–1.73)**	1.40(1.13–1.72)**
**Place of residence**				
Rural		1.00	1.00	1.00
Urban		1.51(1.28–1.77)***	1.42(1.20–1.70)***	1.31(1.09–1.58)**
**Religion**				
Christian		1.00	1.00	1.00
Non-Christian		0.65(0.18–2.34)	0.69(0.19–2.52)	0.66(0.18–2.39)
No religion		1.80(1.10–2.96)*	1.92(1.19–3.10)**	1.88(1.18–2.99)**
**Highest education level**				
No education		1.00	1.00	1.00
Primary		0.94(0.76–1.17)	0.93(0.75–1.16)	0.92(0.74–1.15)
Secondary		1.11(0.82–1.49)	1.02(0.75–1.39)	1.03(0.75–1.41)
Higher		1.04(0.66–1.62)	0.87(0.54–1.41)	1.00(0.61–1.65)
**Literacy level**				
Cannot read at all		1.00	1.00	1.00
Able to read only parts of sentence		0.93(0.74–1.16)	0.91(0.72–1.15)	0.89(0.70–1.13)
Able to read whole sentence		0.78(0.62–0.99)*	0.75(0.58–0.97)*	0.75(0.58–0.96)*
No card with required language		0.60(0.23–1.56)	0.60(0.22–1.60)	0.61(0.23–1.64)
Blind/visually impaired		2.17(0.37–12.75)	1.98(0.35–11.27)	1.89(0.30–12.01)
**Marital status**				
Married		1.00	1.00	1.00
Co-habiting		0.96(0.81–1.14)	0.96(0.81–1.13)	1.01(0.85–1.37)
**Currently residing with partner**				
Living together		1.00	1.00	1.00
Staying elsewhere		0.86(0.70–1.06)	0.86(0.70–1.06)	0.85(0.69–1.05)
**Number of partner’s wives**				
No other wife		1.00	1.00	1.00
1		1.16(0.96–1.39)	1.16(0.96–1.39)	1.14(0.94–1.37)
2		0.96(0.65–1.43)	0.96(0.65–1.43)	1.01(0.68–1.50)
3 or more		1.67(1.16–2.39)**	1.76(1.22–2.53)**	1.70(1.19–2.43)**
Don’t know		1.08(0.66–1.77)	1.07(0.66–1.74)	1.04(0.64–1.68)
**Partner’s age**				
15–24		1.00	1.00	1.00
25–34		0.94(0.70–1.26)	0.96(0.71–1.29)	0.94(0.70–1.28)
35–44		1.07(0.77–1.49)	1.10(0.79–1.54)	1.11(0.80–1.55)
45 +		1.14(0.80–1.64)	1.15(0.80–1.66)	1.14(0.79–1.64)
55 +				
**Partner’s educational level**				
No education		1.00	1.00	1.00
Primary		0.85(0.71–1.03)	0.86(0.71–1.04)	0.91(0.75–1.11)
Secondary		0.94(0.75–1.16)	0.91(0.72–1.14)	0.94(0.74–1.18)
Higher		0.90(0.66–1.24)	0.85(0.62–1.17)	0.91(0.66–1.25)
**Insurance cover**				
No			1.00	1.00
Yes			0.89(0.62–1.28)	0.90(0.63–1.30)
**Internet access**				
No			1.00	1.00
Yes			1.24(0.97–1.57)	1.28(1.00–1.63)
**Ownership of mobile phone**				
No			1.00	1.00
Yes			1.00(0.85–1.18)	1.00(0.85–1.18)
**Watch television**				
No			1.00	1.00
Yes			1.02(0.85–1.24)	1.05(0.86–1.27)
**Listen to radio**				
No			1.00	1.00
Yes			1.18(0.99–1.40)	1.19(1.00–1.42)
**Read newspapers/magazines**				
No			1.00	1.00
Yes			1.05(0.86–1.29)	1.03(0.84–1.27)
**Occupation**				
Not working				1.00
Professional/technical/managerial				0.66(0.45–0.96)*
Clerical				0.45(0.23–0.89)*
Sales				1.22(0.94–1.59)
Agricultural				0.74(0.60–0.91)**
Services				1.07(0.86–1.33)
Manual job				0.49(0.18–1.37)
**Wealth index**				
Poorest				1.00
Poorer				1.05(0.85–1.30)
Middle				0.75(0.59–0.95)*
Richer				1.00(0.79–1.27)
Richest				0.96(0.72–1.28)
**Financial inclusion**				
No				1.00
Yes				1.14(0.94–1.39)

*RR* Relative Risk

^*^p < 0.05, **p < 0.01, ***p < 0.0001

## Discussion

This study examined the association between IPSV and cigarette smoking among women in intimate unions in PNG. The present study adds to the current literature on IPV and cigarette smoking . The study found that women who had experienced IPSV had a greater odds of smoking cigarette. Research on the association between IPSV and health risk behaviors especially cigarette smoking are well documented [20] and consistent with the findings of this study. For instance, in a cross-sectional study that examined the association between intimate partner violence experience and cigarette smoking, Zhang and colleagues [20] found women experiencing intimate partner violence were more likely to smoke cigarette. In the US, a meta-analysis of 31 peer-reviewed studies to evaluate the relationship between intimate partner violence victimization and cigarette smoking revealed victims of intimate partner violence are at greater risk of smoking with a composite side effect of d ¼ 0.41 [21]. Thus, across the collected and analyzed literature, victims of IPV are significantly more likely to engage in smoking behavior than non-victims.

Sexual violence has been found to be more closely linked to activities such as cigarette smoking than other types of intimate partner abuses. Sexual IPV victimization exhibited the most pronounced connections with cigarette smoking, according to a study done to investigate the health status and health risk behaviors related with experiences of psychological, physical, or sexual IPV among women getting care at a medical center [22]. Victims of IPV are more likely to smoke cigarette than offenders of IPV [21]. It is important to also note that because of the circumstances surrounding intimate sexual assault, it has great impact on victims' psyches, and the psychological repercussions last longer [21]. Studies  have shown that victims of IPV experience mental health problems such as depression, generalized anxiety disorder, suicide risk, and post-traumatic stress disorders [23–25], loneliness, sleeping problem and short sleep [26] leading to variety of drug use disorders [24]. These psychological outcomes associated with IPV are indicated to profound in women than in men [25]. Victims of IPV in most circumstances resort to health risk behaviors such as cigarette smoking as coping techniques against the stresses experienced [21]. Women who are victims of IPV also find consolation in smoking when they are unable to report the abuse to family members or law enforcement authorities [9, 11]. Furthermore, victims of IPV are sometimes forced by an abusive partner to use drug substances including smoking cigarette [27]. A survey conducted by a national center on domestic violence, trauma and mental health in the US found many victims of IPV are forced or coerced by abusive partners to use substances [28]. Our findings support the assertion that women are more likely to smoke as a psychological coping mechanism when they suffer stress, anger, or despair due to IPV, and the association between stress and cigarette smoking has been reported to be stronger in women than in men [29].

The present study also provides evidence to demonstrate that certain demographic and socioeconomic positions including age, wealth index, occupation, partner’s number of wives, region, place of residence, religion and literacy play a role in cigarette smoking among women in union. Evidently, when compared to their counterparts, those from the Highlands region, those who live in urban areas, those with no religious affiliation, and those whose partners have three or more wives, had a greater odds of smoking cigarette. Wilson [30] posits that lower IPV is sometimes attributed with marriage, urban residency, and increasing age. Although all women can be victims of IPV regardless of their age, marital status, level of education, income status, place of residence and country of residence [31], Bhona et al. [32] found that women who have greater educational and socioeconomic levels are less likely to be victims of partner violence.

The findings of this study suggest that women between the ages 25–29 years are mostly affected by IPSV and engaged in cigarette smoking as compared with other age group. This supports the assertion that IPSV affects people of all socioeconomic backgrounds, but youth from lower socioeconomic backgrounds are more likely to be exposed and suffer [24]. Moreover, women of lower and higher socioeconomic status, have more cigarette smoking tendencies as compared to those of middle socioeconomic status [33] as suggested by the findings of this study. Additionally, the findings corroborate with a study which revealed that women with no formal education, primary-level, or secondary-level qualification have a larger chance of being smokers than women with a higher education [34]. Perhaps, women with high education are more likely to have accessed information on the negative consequences of smoking.

Some strengths associated with the present study need to be remarked. The study utilized a nationally representative data to examine the association between IPSV and cigarette smoking in PNG, thereby increasing the generalizability of its findings. Methodologically this study is also associated with some strengths. The present study uses a relatively new analytical approach by applying the modified Poisson regression that incorporates the robust error variance procedure to establish the association between IPSV and cigarette smoking. The modified Poisson regression approach can be regarded as very reliable in terms of both relative bias and percentage of confidence interval coverage [18]. Also, extensive discussion in much of the literature has reached a consensus that the relative risk is preferred over the odds ratio for most prospective studies with binary outcomes as logistic regression modelling overestimates the odds ratios [18, 35–38]. In that regard, the use of Poisson regression has been a promising alternative. Of course, there are some limitations that need to be commented. Our study does not explore any causal relationship between IPSV and smoking, as PNGDHS data are cross-sectional. In addition, the present study relied on self-reported data which may be subjected to recall bias. Even accurate self-reported measures may reflect individual differences not associated with health per se. Also, the IPSV variable was collected through an optional domestic violence module and as a result such type of sensitive information could not be reported or misreported by the participants. Moreover, in this study current cigarette smoking was defined as smoked cigarette in the last 24 h before the survey. This could have the potential to exclude women with a non-daily smoking pattern. Despite these limitations, the research presented here is suggestive and represents important progress. It calls attention to an association with scarce empirical examination in part due to limited research especially in the context of PNG.

### Public health and policy implications

This study offers a number of policy implications that need to be acknowledged. For the purpose of this study, implications of the study have been grouped into three key areas; 1) public health and practice implications 2) health policy implications 3) research implications. First of all, in relation to the public health and practice implications, gender-based institutions and groups in collaboration with the PNG National Department of Health should organize health education and awareness creation campaign on cigarette smoking and IPSV in PNG. The health education and awareness intervention should primarily center on health, social and economic risks associated with smoking cigarette among women who experience IPSV. Based on the findings of this study, the health education program should target more of women from the Momase region, those residing in urban area and those whose partners have three or more wives since they were having a higher log count on cigarette smoking in PNG. Also, the proposed health campaign should target men and further educate them on the need not to expose women to IPSV because of the dangers associated with it. Again, since our findings showed that cigarette smoking and IPSV are significantly associated, increasing health campaign against IPSV could scale down cigarette smoking. As part of this health campaign, gender-based institutions and groups in collaboration with PNG health institutions should have a panel discussion with both men and women on why men expose women to IPSV. To the best of our knowledge, this form of discussion on experience of IPSV among women would help provide a framework to guide public health education on IPSV and its association with cigarette smoking. Concerning the health policy implications, we propose that the development/formulation of health policy that aims to reduce cigarette smoking among women who experience IPSV should include other significant demographic, social and economic variables such as region of residence, place of residence (rural/urban), religion, number of wives of partners, age, wealth index and nature of employment. This is because aside from IPSV, the above significant factors play a major role in cigarette smoking among women in PNG. Lastly, in terms of the research implications, since this study did not look at the following important research areas due to the nature of the dataset employed in this study, future research should investigate the following areas; 1) perpetuators of IPSV and associated factors; 2) knowledge of women experiencing IPSV on the health, social and economic risks associated with cigarette smoking; 3) the moderating role of self-rated health in the association between experience of IPSV and cigarette smoking among women; 4) enablers/facilitators of cigarette smoking among women experiencing IPSV. Such studies can provide a comprehensive understanding of IPSV, cigarette smoking and the association between these experiences capable of influencing policies and interventions.

## Conclusion

The rates of IPSV and cigarette smoking among women in PNG in the current study were relatively high. IPSV is shown to be a significant predictor of cigarette smoking among women in union in PNG. Understanding this association suggests that policymakers and relevant authorities in PNG can act to address IPSV to reduce cigarette smoking. Counseling to help victims of IPSV cope with stresses, awareness creation, service provision and program design on intimate partner violence are urgently required to minimize cigarette smoking among women in union in PNG.

## Supplementary Information

**Additional file 1.**

## Data Availability

The data that support the findings of this study are available from the DHS. However, restrictions apply to the availability of the data, which were used under license for the current study; thus, the data are not publicly available. However, they can be made available from the authors upon reasonable request with the permission of DHS programs.

## Abbreviations

IPVS: Intimate partner sexual violence
PNG: Papua New Guinea
IPV: Intimate Partner Violence
CI: Confidence Intervals
PNGDHS: Papua New Guinea Demography and Health Survey
DHS: Demographic and Health Survey
ICF: Inner City Fund
UNFPA: United Nations Population Fund
CUs: Census Units
