# Supplementary material for: Association between experiences of intimate partner sexual violence and cigarette smoking among women in union in Papua New Guinea: evidence from a nationally representative survey
Source: BMC Public Health. 2022 Mar 29;22:613. doi: 10.1186/s12889-022-13003-4 (PMC8966284; doi:10.1186/s12889-022-13003-4)
Supplement: Supplementary file 1 — Additional file 1. [file 12889_2022_13003_MOESM1_ESM.docx]

**Association between experiences of intimate partner sexual violence and cigarette smoking among women in union in Papua New Guinea: Evidence from a nationally representative survey**

**Bernard Yeboah-Asiamah Asare^a^, Williams Agyemang-Duah^b^, Emmanuel Brenyah Adomako^c^ Parul Puri^d^, Deborah Odunayo Ogundare^e^, Deepanjali Vishwakarma^f^, Prince Peprah^g^**

^a^ Curtin School of Population Health, Faculty of Health Sciences, Curtin University, Kent Street, Perth, Australia, [bernard.yeboah@postgrad.curtin.edu.au](mailto:bernard.yeboah@postgrad.curtin.edu.au)

^b^ Department of Geography and Planning, Queen’s University, Kingston, Ontario, K7L 3N6, Canada; [agyemangduahwilliams@yahoo.com](mailto:agyemangduahwilliams@yahoo.com)

^c^ Social Work Department, School of Health and Society, University of Wollongong, Wollongong, Australia; [eab673@uowmail.edu.au](mailto:eab673@uowmail.edu.au)

^d^ Department of Mathematical Demography and Statistics, International Institute for Population Sciences, Mumbai-400088, Maharashtra, India, [parulpuri93@gmail.com](mailto:parulpuri93@gmail.com)

^e^ Nigeria Centre for Disease Control, Abuja, Nigeria, [oyewaledeborah1995@gmail.com](mailto:oyewaledeborah1995@gmail.com)

^f^ Department of Mathematical Demography and Statistics, International Institute for Population Sciences, Mumbai-400088, Maharashtra, India, [deepanjali.vishwakarma7@gmail.com](mailto:deepanjali.vishwakarma7@gmail.com)

^g^Centre for Primary Health Care and Equity/ Social Policy Research Centre, University of New South Wales, Sydney, Australia; [p.peprah@unsw.edu.au](mailto:p.peprah@unsw.edu.au)

**Correspondence to:** Parul Puri, Department of Mathematical Demography and Statistics, International Institute for Population Sciences, Mumbai-400088, Maharashtra, India, [parulpuri93@gmail.com](mailto:parulpuri93@gmail.com)
